# Current Status and Complexity of Three *Begomovirus* Species in Pepper Plants in Lowlands and Highlands in Java Island, Indonesia

**DOI:** 10.3390/v15061278

**Published:** 2023-05-30

**Authors:** Andi Wahyono, Rudi Hari Murti, Sedyo Hartono, Tri Rini Nuringtyas, Arman Wijonarko, Mulyantoro Mulyantoro, Deni Firmansyah, Ahmad Afifuddin, Innez Candri Gilang Purnama

**Affiliations:** 1 Department of Horticulture Crop Research Development, PT BISI International Tbk, Kediri 64293, Indonesia; andi.wahyono@bisi.co.id (A.W.); mulyantoro@bisi.co.id (M.M.); deni.firmansyah@bisi.co.id (D.F.); 2 Department of Agronomy, Faculty of Agriculture, Universitas Gadjah Mada, Sleman 55281, Indonesia; 3 Department of Plant Protection, Faculty of Agriculture, Universitas Gadjah Mada, Sleman 55281, Indonesia; sedyohartono@ugm.ac.id (S.H.); arman_w@ugm.ac.id (A.W.); 4 Department of Tropical Biology, Faculty of Biology, Universitas Gadjah Mada, Sleman 55281, Indonesia; tririni@ugm.ac.id; 5 Department of Biotechnology, PT BISI International Tbk, Kediri 64175, Indonesia; ahmad.afifuddin@bisi.co.id (A.A.); innez.gilang@bisi.co.id (I.C.G.P.)

**Keywords:** *Begomovirus*, *Bemisia tabaci*, mixed infection, PepYLCIV, virus diversity

## Abstract

Three primary species from the *Begomovirus* genus, *Pepper yellow leaf curl Indonesia virus* (PepYLCIV), *Tomato yellow leaf curl Kanchanaburi virus* (TYLCKaV), and *Tomato leaf curl New Delhi virus* (ToLCNDV), are suspected of spreading throughout pepper production centers, and plants are infected by a single species or a combination of two or three species. This study was conducted to provide complete information about the symptoms, incidence and severity, whitefly biotypes, as well as the dominance status of the three *Begomovirus* species in pepper-producing areas in Java. A DNA analysis was carried out on leaf samples to identify *Begomovirus* species and biotypes of *B. tabaci* collected from 18 areas (16 districts) in lowlands (<400 m asl) and highlands (>700 m asl). The DNA analysis showed that *B. tabaci* biotype B was the most commonly detected in all locations compared to the A, AN, and Q biotypes. The incidence of begomovirus infection was at a high level, 93% and 88.78% in the lowlands and highlands, respectively. However, the severity of begomovirus infection was significantly higher in the lowlands (54.50%) than in the highlands (38.11%). A single infection of PepYLCIV was most dominant in all locations sampled and caused severe infection, followed by a mixed infection with TYLCKaV. Therefore, the current status of begomovirus infection, especially PepYLCIV, can provide advice to farmers using more tolerant and resistant varieties as well as a breeding strategy for resistant pepper varieties.

## 1. Introduction

Pepper (*Capsicum annuum*) is one of the most important vegetable commodities in the world, including the Indonesia market. The demand for pepper in the market is increasing but is constrained by an unstable production caused by the rainy season and biotic stress. Regarding biotic stress, *begomovirus* infection is the main obstacle factor for pepper production [1,2], especially in drought season. Based on Statistics Indonesia data, chili pepper production has tended to decrease over the last 5 years, from 2017 to 2021. Chili pepper production in 2021 was 1.39 million tons, a 8.09% (121.96 thousand tons) decrease from the 2020 production [3]. The *Begomovirus* genus causes severe diseases in major vegetable crops such as pepper, especially in the tropics and subtropics of Asia and America. Diseases caused by geminiviruses (family *Geminiviridae*; genus *Begomovirus*) are unquestionably the most severe and devastating among others [4]. *Begomovirus* is one of the genera in the Geminiviridae family that has the most species, more than 320 species [5]. Begomoviruses have been reported to contain monopartite and bipartite genomes [6]. *Begomovirus* have circular, single-stranded DNA genomes that replicate through double-stranded intermediates in the nuclei of infected plant cells. Viral double-stranded DNA also assembles into minichromosomes and is transcribed in infected cells.

Begomoviruses are transmitted persistently by members of the *Bemisia tabaci* species complex [7]. Long-distance spreading by infested plant materials or wind currents contribute to their status as one of the world’s most economically important agricultural pests [8]. The differential transmission depends on the *B. tabaci* biotype [9]. Fiallo-Olivé et al. [9] explained the variance of several *B. tabaci* biotypes, namely biotype A as a new species (the Americas), the B biotype as a Middle East-Asia Minor 1 (MEAM1) species, the Q biotype as a Mediterranean species (MED), and the other biotypes as Asian species. In China, the B biotype has rapidly spread to more than ten provinces and is more malignant in damaging a variety of vegetables other than the non-B biotype. Currently, the *B. tabaci* biotype B is able to breed its population twice faster than non-B biotypes [10]. The existence of *B. tabaci* biotype B requires caution because it has the potential to be more dangerous than non-B biotypes [11].

The common symptoms of begomovirus infection are yellowing, leaf curling or rolling, stunting, and a reduced fruit yield per plant. The *Begomovirus* genus that infects pepper plants can cause a decrease in yield loss by up to 100% [12]. In the field, symptoms of begomovirus infection on pepper are very typical such as chlorosis, mosaic, vein clearing, and bleaching [13]. Viral infections such as yellow curly leaves on pepper plants have spread rapidly into central pepper plants in Yogyakarta, Bali, and Nusa Penida [14,15,16]. The emergence of the first infection of *Begomovirus* disease in Indonesia is supported by evidence of *B. tabaci* mainly to two species of the complex, MEAM1 (Middle East–Asia Minor I, formerly referred to as biotype B) and Med (Mediterranean, formerly referred to as biotype Q), species that are highly invasive, highly polyphagous, and that transmit viruses on various crop [17,18]. Begomovirus infection initially occurred in several pepper-producing areas with a limited range of areas, in the lowlands, with hot temperatures and an intensive infection in the dry season.

However, the development of infection areas is expanding not only in the lowlands but in the highlands, both in the dry and rainy seasons. This indicates an increase in virulence and an increase in the adaptability of the virus. In Indonesia, there are three main species of the *Begomovirus* genus that infect pepper plants, i.e., *Pepper yellow leaf curl Indonesia virus* (PepYLCIV), *Tomato yellow leaf curl Kanchanaburi virus* (TYLCKaV), and *Tomato leaf curl New Delhi virus* (ToLCNDV). Comprehensive information about the symptoms, incidence, and severity, as well as the dominance of the spreading area, of the three *Begomovirus* species in pepper-producing areas in Java as the main production area was urgently needed. Therefore, the objective was to conduct mapping activities of the three *Begomovirus* species to provide advice to the farmers when planting pepper varieties based on the resistance status of the varieties and the severity of the *Begomovirus* infection in their area. This research was conducted to provide comprehensive information about the symptoms, incidence, and severity, as well as the status dominance, of the three *Begomovirus* species in pepper-producing areas in Java.

## 2. Materials and Methods

### 2.1. Field Survey and Samples Collection

**Figure 1:**
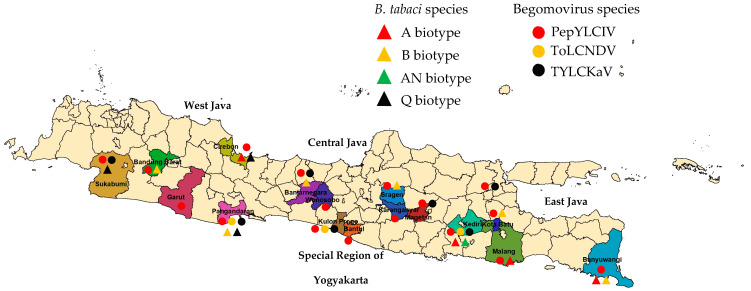
Sampling locations in Java to collect pepper-infected begomovirus. Lowlands (<400 m asl): West Java: Cirebon and Pangandaran; Central Java: Sragen; East Java: Kediri and Banyuwangi; Special Region of Yogyakarta: Bantul and Kulon Progo); highlands (>700 m asl): West Java: Sukabumi, Garut, and Bandung Barat; Central Java: Banjarnegara, Wonosobo, and Karanganyar); East Java: Malang, Batu, and Magetan).

A Survey was conducted from June to October 2022 from several pepper-producing center areas (18 areas, 16 districts) in lowlands (<400 m asl) and highlands (>700 m asl) in Java, Indonesia (Figure 1). Samples were taken with a purposive sampling according to typical begomovirus symptoms (yellowing, mosaic, leaf curl, smaller leaf, cupping, and stunting). Twenty samples were taken from each area that represented each typical symptoms, thus the number of samples was 360. A collection of vector insects *B. tabaci* was also conducted in each area. Leaf samples and whiteflies were collected for a DNA analysis. At the same time, disease incidence (%) and disease severity (%) were determined by counting and scoring the number of plants that showed symptom from 5% of the plant population according to a systematic random sampling. However, disease severity was assessed based on a modified scale of category according to Adilah and Hidayat [19]. Symptom development was evaluated according to the following scale: 0, no visible symptoms; 1, yellow leaves; 2, yellow and curly leaves, 3; yellow, mosaic, and leaf curl; 4. yellow, mosaic, curly, cupping, and stunting.

Disease severity = (∑ (ni × vi)/(Z × V)) × 100%

where ni is the number of infected plants in the same category; vi is the severity score; Z is the maximum rating score; and V is the total number of plants observed.

### 2.2. DNA Extraction and Identification of Begomovirus Species and Bemisia tabaci Biotypes

The leaves of 20 samples were taken from each area and the 360 samples were analyzed for identifying begomovirus species. A total DNA isolation from the leaf samples was conducted following the method described by Lukman et al. [20]. For whitefly samples, the genomic DNA of each sample was extracted using Geneaid Genomic DNA Mini Kit (Tissue) Protocol (https://geneaid.com/data/files/1605685391109197921.pdf, accessed on 30 June 2022).

PCR reaction mixtures of begomovirus species identification were set up in a total volume of 10 μL containing 20 ng of template, 1X DreamTaq Buffer (containing 1.5 mM MgCl_2_), 0.2 mM dNTP mix, 0.25 µM each of forward and reverse primers, and 1 U of Dream Taq DNA Polymerase (Thermo Fisher Scientific, Waltham, MA, USA). The PCR condition was carried out with a predenaturation at 94 °C for 4 min followed by 35 cycles of denaturation at 94 °C for 30 s, an appropriate annealing primer for 1 min, an extension at 72 °C for 1.5 min, and a final extension at 72 °C for 7 min. The PCR identification of whitefly biotypes was set up in a total volume of 12.5 μL containing 20 ng of template, 1X My Taq HS Red Mix 2x Bioline (Meridian Bioscience, Cincinnati, OH, USA), 3.5 mM MgCl_2_, and 0.2 µM each of forward and reverse primers. The PCR reaction was carried out with a predenaturation at 94 °C for 5 min followed by 35 cycles of denaturation at 94 °C for 1 min, an appropriate annealing primer for 1 min, an extension at 72 °C for 1 min, and a final extension at 72 °C for 5 min.

Amplifications were performed on a SimpliAmp Thermal Cycler–Applied Biosystems (Thermo Fisher Scientific, Waltham, MA, USA). The PCR was carried out using a primer set according to Table 1 for the detection of three species of begomovirus, i.e., *Pepper yellow leaf curl Indonesia virus* (PepYLCIV), *Tomato yellow leaf curl Kanchanaburi virus* (TYLCKaV), and *Tomato leaf curl New Delhi virus* (ToLCNDV). All amplicons were then separated by electrophoresis in 1.5% agarose gel, stained with FluoroVue™ Nucleic Acid Gel Stain (Smobio), and visualized on Gel Doc (Kodak MI, Rochester, NY, USA) to evaluate the PCR amplification.

### 2.3. Statistical Analysis

All data on disease incidence and severity and percentage of virus-infected plants were subjected to an independent-sample *t*-test analysis with the SPSS program version 25.

## 3. Results

### 3.1. Disease Symptoms, Incidence, and Severity

Our survey was conducted in 18 pepper-producing areas covering 16 districts in West Java, Central Java, East Java, and the Special Region of Yogyakarta from June to October 2022 (Figure 1). Most samples were taken from pepper plant populations of various varieties, which showed a susceptibility to infection with typical symptoms of begomovirus. In general, symptoms of begomovirus infection in pepper plants were more varied and more severe in the lowlands than in the highlands (Table 2).

The incidence of begomovirus infection in the lowlands and highlands ranged from 82% to 100% and 69% to 95%, respectively (Table 2). On average, the incidence of begomovirus infection was higher in the lowlands, i.e., 93.00%, than in the highlands, i.e., 88.78%, but not significantly different (t = 1.26; P = 0.23). Meanwhile, the severity of begomovirus infection was significantly higher in the lowlands, i.e., 54.50%, than in the highlands, i.e., 38.11% (t = 2.57; P = 0.02).

### 3.2. Biotypes of B. tabaci

The result of the DNA analysis of *B. tabaci* identified four biotypes (A, B, AN, and Q) (Figure S1). Eight samples were included in the B biotype, four samples for each of the A and Q biotype, and one sample was included in the AN biotype (Table 3). In the lowland areas, we found *B. tabaci* with a single biotype in seven areas and a double biotype in two areas, while in the highland areas, we found a single biotype or even no *B. tabaci* found in three of the nine areas. In the locations of Garut-West Java and Wonosobo-Central Java, *B. tabaci* was not found during our observations, but another species of whitefly was found, namely *Trialeurodes vaporariorum*. The results of the DNA analysis of *B. tabaci* did not find any Nauru and S biotypes in all mapping areas.

### 3.3. Identification of Three Begomovirus Species

The identification results of the 360 samples showed that 329 samples were infected with begomovirus, and 31 samples were not (Table 4). Based on a PCR analysis, the infection percentage of begomovirus in the lowlands, i.e., 90.00%, was not significantly different than in the highlands, i.e., 92.78% (t = 0.40; P = 0.69). PepYLCIV was detected in all observed samples, followed by TYLCKaV and ToLCNDV (Figure S2). Mixed infections of PepYLCIV and TYLCKaV were more frequent than with ToLCNDV. A mixed infection of three species was only found in two locations (Kediri-East Java and Kulon Progo-Yogyakarta).

Based on the species composition, a single PepYLCIV infection had the same average incidence in the lowlands and highlands, but the average severity was higher in the lowlands than in the highlands. Cases of double and triple mixed infection of PepYLCIV–ToLCNDV–TYLCKaV also contributed to the increased disease severity level of 81.25% in Kediri-East Java in the lowlands.

### 3.4. Phenotypic Symptoms of Single and Mixed begomovirus Infection

**Figure 2:**
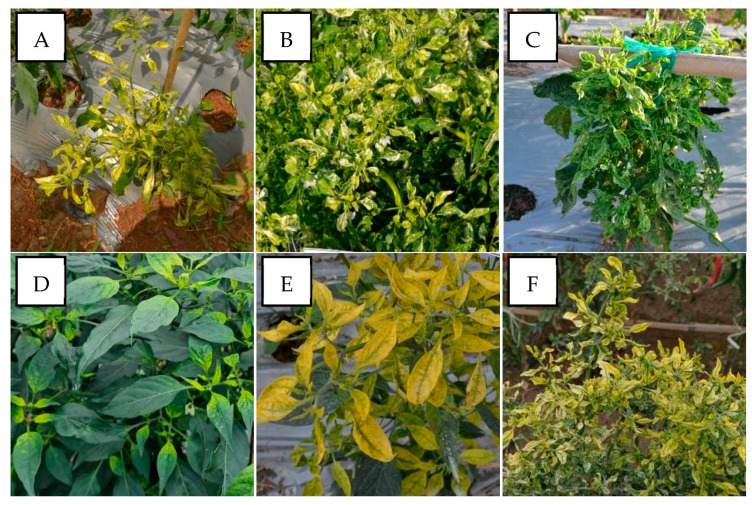
Variation in the symptoms of pepper plants infected by PepYLCIV in single infections in lowlands and highlands verified by a PCR detection. (**A**) Yellowing, leaf curl, and cupping in Pangandaran 1-West Java (lowland); (**B**) yellowing, curling, cupping, vein banding, and leaf of reduced size in Bantul-Yogyakarta (lowland); (**C**) green mosaic, curling, cupping, vein banding, leaf of reduced size, and stunting in Kediri-East Java (lowland); (**D**) chlorosis symptom in Sukabumi-West Java (highland); (**E**) yellowing and leaf cupping upward in Banjarnegara-Central Java (highland); (**F**) yellowing and leaf curl in Malang-East Java (highland).

**Figure 3:**
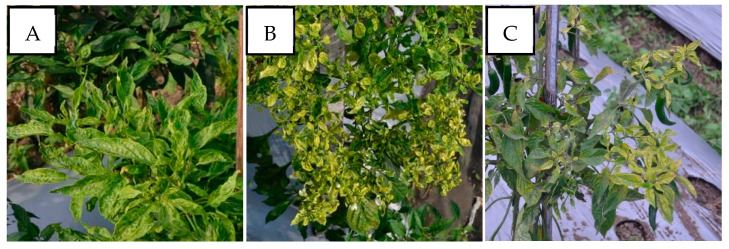
Variation in the symptoms of pepper plants infected by TYLCKaV in single infections in lowlands and highlands verified by a PCR detection. (**A**) Yellow mosaic, leaf curl, and vein banding (Kulon Progo-Yogyakarta); (**B**) yellowing, leaf curl, cupping upward and downward, vein banding, and leaf of reduced size (Kulon Progo-Yogyakarta); (**C**) yellowing, vein banding, and leaf of reduced size (Magetan-East Java).

PepYLCIV appeared to be the major begomovirus infecting pepper as a single infection and as mix of double and triple infections. The most commonly observed symptoms were yellowing, yellow and green mosaic, leaf curl, and leaf cupping. The symptoms of a PepYLCIV infection in the lowlands were yellowing, green mosaic, leaf curl, leaf of reduced size, cupping, vein banding, and stunting (Figure 2A–C), whereas the symptoms in highland areas were chlorosis, yellowing, leaf curl, and leaf cupping (Figure 2D–F). From this observation, the symptoms of PepYLCIV infection in the lowlands were observed to be more severe than in the highlands, causing the leaves to be yellow with mosaic, leaf of reduced size, leaf cupping, and severe stunting of the plants. Variations in the symptoms of pepper plants infected with TYLCKaV included yellowing, yellow mosaic, leaf curl, leaf of reduced size, cupping upward and downward, and vein banding (Figure 3).

**Figure 4:**
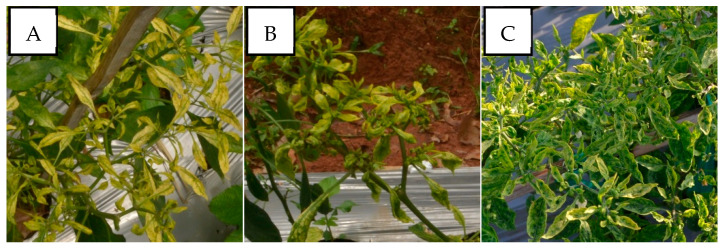
Variation in the symptoms of pepper plants infected by PepYLCIV–ToLCNDV in mixed infections in lowlands verified by a PCR detection. (**A**) Yellow mosaic, leaf curl, and vein banding (Pangandaran 1-West Java); (**B**) yellow mosaic, leaf curl, and leaf of reduced size (Pangandaran 1-West Java); (**C**) green mosaic, leaf curl, cupping upward, vein banding, and leaf of reduced size (Kediri-East Java).

**Figure 5:**
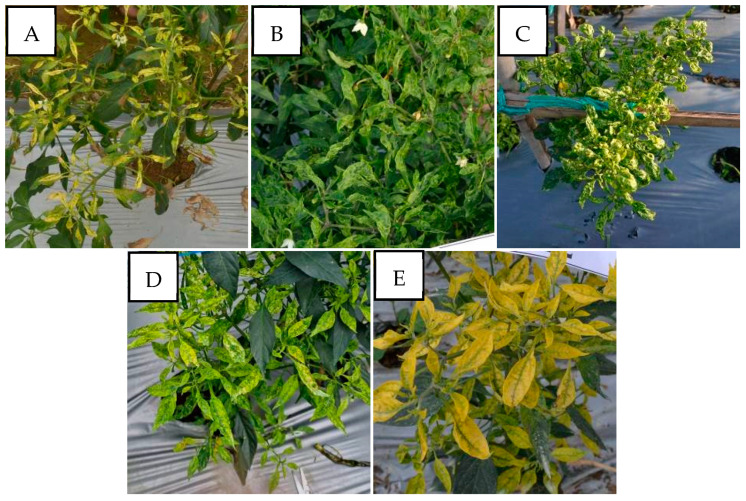
Variation in the symptoms of pepper plants infected by PepYLCIV–TYLCKaV in mixed infections in lowlands and highlands verified by a PCR detection. (**A**) Yellowing, leaf curl, and leaf of reduced size (Pangandaran 2-West Java); (**B**) green mosaic, leaf curl, and leaf of reduced size (Kulon Progo-Yogyakarta); (**C**) yellow mosaic, leaf curl, cupping downward, vein banding, leaf of reduced size, and stunting (Kediri-East Java); (**D**) yellow mosaic and vein banding (Sukabumi-West Java); (**E**) yellowing, vein banding, and cupping upward (Banjarnegara-Central Java).

**Figure 6:**
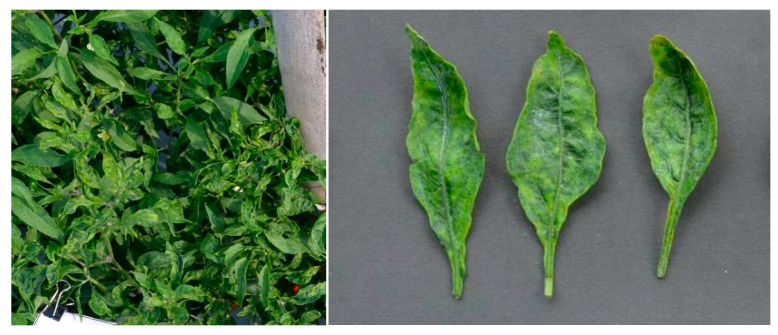
Symptoms of a pepper plant and leaves infected by ToLCNDV–TYLCKaV in mixed infections in lowland Kulon Progo-Yogyakarta. The symptoms showed yellow mosaic, leaf curl, vein banding, and cupping upward.

**Figure 7:**
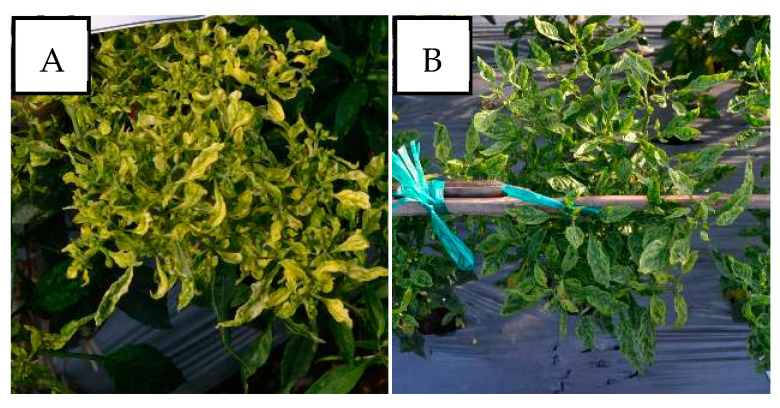
Variation in the symptoms of pepper plants infected by PepYLCIV–ToLCNDV–TYLCKaV in mixed infections in lowlands verified by a PCR detection. (**A**) Yellowing, leaf curl, vein banding, cupping upward, and leaf of reduced size (Kulon Progo-Central Java); (**B**) yellow mosaic, leaf curl, vein banding, cupping upward, and stunting (Kediri-East Java).

In cases of mixed infections, PepYLCIV–ToLCNDV showed yellow and green mosaic, leaf curl, leaf of reduced size, leaf cupping upward, and vein banding (Figure 4). The symptoms of PepYLCIV–TYLCKaV infection were yellowing, yellow and green mosaic, leaf curl, leaf of reduced size, cupping upward and downward, vein banding, and stunting (Figure 5). Meanwhile, the symptoms of ToLCNDV–TYLCKaV infection in Kulon Progo-Yogyakarta showed yellow mosaic, leaf curl, vein banding, and cupping upward (Figure 6). The symptoms of PepYLCIV–ToLCNDV–TYLCKaV infection were observed in pepper plants in Kulon Progo-Central Java and Kediri-East Java and showed yellowing, yellow mosaic, leaf curl, vein banding, cupping upward, leaf of reduced size, and stunting (Figure 7). In this survey, ToLCNDV was not found in a single infection but in a mixed infection with PepYLCIV and TYLCKaV.

**Figure 8:**
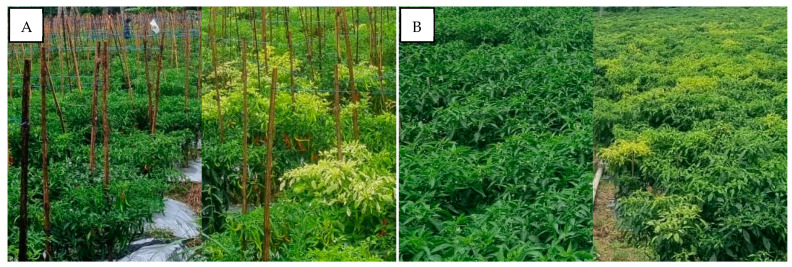
Performance comparison of pepper plant population infected by begomovirus and asymptomatic resistant pepper plants in two mapping areas. (**A**) Sukabumi-West Java in the highlands; (**B**) Kulon Progo-Yogyakarta in the lowlands.

**Figure 9:**
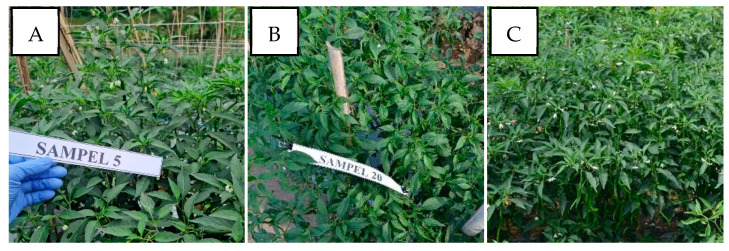
Resistant pepper varieties showed asymptomatic performance but were confirmed positive for begomovirus infection and verified by PCR detection. (**A**) Sios Tavi F1 variety infected PepYLCIV in highland Sukabumi-West Java; (**B**) Sios Tavi F1 variety infected TYLCKaV in lowland Kulon Progo-Yogyakarta; (**C**) Iggo Tavi F1 variety infected PePYLCIV in lowland Bantul-Yogyakarta.

In this survey, the pepper plant populations were also found asymptomatic of infection near the observed susceptible pepper varieties. In Sukabumi-West Java in the highlands and Kulon Progo-Yogyakarta in the lowlands, the performance comparison of susceptible varieties to begomovirus infection remained symptomatic, and resistant varieties showed no symptoms (Figure 8). The pepper plants with genetic resistance showed an asymptomatic performance but a confirmed positive begomovirus infection. The pepper varieties were Sios Tavi (PT. BISI International Tbk, Kediri, Indonesia) in Sukabumi-West Java in the highlands and Kulon Progo-Yogyakarta in the lowlands and Iggo Tavi (PT. BISI International Tbk, Kediri, Indonesia) in Bantul-Yogyakarta in the lowlands (Figure 9). Sios Tavi and Iggo Tavi are new F1 hybrid pepper varieties which are resistant to begomovirus, from PT. BISI International, Tbk Indonesia.

## 4. Discussion

The incidence, severity, and spreading of begomovirus infection were determined by whitefly–begomovirus interaction. Although there was no significant incidence of begomovirus infection between lowland and highland areas (Table 2), the result of the mapping of 18 pepper-producing areas in Java showed a high incidence of begomovirus infection in both lowlands and highlands, 93% and 88.78% on average, respectively. This result was higher than the incidence of begomovirus infection in Myanmar (59.3%) in pepper and tomato plants [24]. The Java region’s extremely high incidence rate demonstrates the pepper’s vulnerability to the begomovirus. By eradicating the population of *B. tabaci*, which serves as a begomovirus vector, efforts can be made to reduce this incidence. Another attempt to reduce a virus infection of pepper was by a rotation with nonhost crops for *B. tabaci* or other viruses.

The severity of the begomovirus infection was significantly higher in the lowlands, i.e., 54.50%, than in the highlands, i.e., 38.11%. Our result showed that PepYLCIV was the main cause of severe infection in single and mixed infections (Table 4). It was similar to results from Annisaa et al. [14] and Fadhila et al. [25], who reported that PepYLCIV was the dominant species and was found to infect plants alone or mixed with other species. In single infections, PepYLCIV was detected in 17 locations with varied severity and symptoms (Figure 2). Meanwhile, single TYLCKaV infections were only detected in two locations at Kulon Progo-Yogyakarta and Magetan-East Java with less severity and symptoms (Figure 3). The same result was shown by Subiastuti et al. [26]; they reported that TYLCKaV was dominantly infected pepper plants in Kulon Progo-Yogyakarta.

The incidence of mixed infections (44% of nine locations) in the lowlands was higher than in the highlands (22%). A special case was in Kulon Progo-Yogyakarta, where single infections were TYLCKaV infections without PepYLCIV, which were common in other areas. However, mixed infections in Kulon Progo were highest compared to others. PepYLCIV infected plants with one or two other virus species. Unfortunately, severe symptoms were shown in cases of mixed infections, PepYLCIV–ToLCNDV (Figure 4), PepYLCIV–TYLCKaV (Figure 5), ToLCNDV–TYLCKaV (Figure 6), and PepYLCIV–ToLCNDV–TYLCKaV (Figure 7). Similar results were found by Sidik et al. [27], who reported that mixed infections of PepYLCV, TYLCV, ToLCV, and MYMV seemed to be associated, triggering more severe symptoms than a single infection in common bean in East Java. A mixed infection of PepYLCIV, TYLCKaV, and AYVV has been reported to cause severe symptoms and is associated with serious virus problems in pepper production in Northern Sumatera [28].

The result of the studied biotypes (Table 3) indicated the *B. tabaci* B biotype was most commonly found as the vector of begomovirus compared to the A, AN, and Q biotypes at all locations. This result was similar to that of Yao et al. [29], who found the B biotype predominated between 2005 and 2014 in the Fujian province in China. The incidence, severity, and spreading of the begomovirus infection were determined by the whitefly–begomovirus interaction. Fiallo-Olivé et al. [9] and Laarif et al. [30] reported that the B and Q biotypes were invasive biotypes and showed a better ability to spread begomovirus compared to other biotypes and preferred host plants in the families Cucurbitaceae dan Solanaceae. The B and Q biotypes are also known as highly polyphagous, can invade indigenous whitefly and rapidly develop pesticide resistance [31], and have a high level of insecticide resistance [29]. Research from Wei et al. [32] found that TYLCV was also transmitted by B and Q biotypes with the same efficiency.

In three locations, such as in Sukabumi, Wonosobo, and Karanganyar (Table 3), far apart from each other, we did not find *B. tabaci*. According to Kil et al. [33] and Kothandaraman et al. [34] begomoviruses are seed-borne, which increases the chance of long-distance spread. Another species of whitefly, *T. vaporariorum,* was found in Sukabumi, Wonosobo, and Karanganyar during our observations. The dominance of *T. vaporariorum* in the highlands is supported by a broad adaptation of all growth phases at low temperature compared to *B. tabaci* [35]. The data indicated that favorable conditions for *B. tabaci* and *T. vaporariorum* are dominantly determined by the agroclimatic and agricultural system. High daily temperatures and intense solar radiation also have an impact on reducing plant resistance to a begomovirus infection, as this condition is suitable for increasing the whitefly population and directly affects the increase of mixed infections rather than single infections. As stated by Hidayat and Rahmayani [36], those increases in infection and symptom severity are highly correlated with whitefly populations, especially during long summer periods. Moreover, they are also caused by the possibility the begomovirus is transmitted by vectors other than *B. tabaci*, i.e., by *T. vaporariorum*, since the results of previous studies showed that ToLCNDV was transmitted by *T. vaporariorum* in India [9,37].

The high *B. tabaci* population in the field was followed by a high incidence of PepYLCIV disease, which also led to a high percentage of *B. tabaci* carrying begomovirus. This showed that the whitefly vector was the main cause of begomovirus transmission in the field [38]. The incidence and severity of the disease were found to be more severe in plantations with monoculture planting patterns compared to those with the intercropping pattern [15]. The interaction synergy among species of begomovirus that infects pepper has been reported to be the cause of the breakdown of the natural defense system of pepper plants [39,40]. Based on this fact, it is interesting to note that it is necessary to avoid continuous planting of one family of plants in an area to avoid a mixed infection of begomovirus which can cause a breakdown of the resistance of a plant.

The other factor contributing to PepYLCIV being the major species of begomovirus that infected pepper compared to TYLCKaV and ToLCNDV was its capacity for infection and a more efficient and strong spread compared to other species. The ability to infect and transmit PepYLCV was related to its very quick mutation ability. This resulted in the emergence of a variety of symptoms in the field and also a molecular diversity of PepYLCIV [41]. Furthermore, virus strains are continuously evolving from monopartite to bipartite species, which may overcome the previous resistance [6]. The *Begomovirus* mutation from the monopartite genome to the bipartite one (A and B) has further been explained. Monopartite to bipartite genome changes result in a higher ability to infect and spread. The research results by Ouattara et al. [42] indicated that the DNA-B component of PepYVMLV caused an increased virulence associated with a higher accumulation of viral DNA in plant tissues, an increased number of contaminated nucleic acids from the phloem parenchyma, and a higher transmission rate by *B. tabaci*. PepYLCV is a disease caused by a serious threat to the pepper production in many regions in the world, including in Indonesia [2,12,28].

In this survey, some pepper plants were also found not to show any phenotypic symptoms of infection, even though they were planted near to the observed susceptible pepper varieties. The asymptomatic pepper plants were F1 hybrid varieties, namely Sios Tavi (PT. BISI International Tbk, Kediri, Indonesia) in Sukabumi-West Java (highlands) and Iggo Tavi (PT. BISI International Tbk, Kediri, Indonesia) in Kulon Progo-Yogyakarta (lowlands). Sios Tavi and Iggo Tavi were positively infected by PepYLCIV and TYLCKaV as found via a PCR detection. This phenomenon indicated that the variety has a genetic resistance to begomoviruses. Based on observations, the pepper plants showed no symptoms of infection, grew normally, and fruit production and yield were still optimum. The use of susceptible or tolerant varieties with proper cultivation and the prevention of whitefly populations were not enough to avoid begomovirus infection. Therefore, alternative methods to effectively control this virus should use resistant varieties. With the use of resistant varieties, even though the begomovirus still infects, it cannot carry out any replication activity in the host cells. Resistant plants can control viruses by preventing the systemic spread of viruses and preventing viral gene (DNA) expression to produce proteins that damage physiological mechanisms [43].

## 5. Conclusions

As the vector of begomovirus, *B. tabaci* biotype B was the most commonly detected biotype compared to the A, AN, and Q biotypes in all locations. Based on our observations, the incidence of begomovirus infection was at the same level, but the severity was significantly higher in the lowland than in the highland areas. Single infections of PepYLCIV were the most dominant ones in all sampled locations and caused severe infections, followed by mixed infections with TYLCKaV. PepYLCIV and TYLCKaV were also found to infect pepper plants of resistant varieties with no symptoms of infection, growing normally, and whose fruit production and yield were still optimum.

## Figures and Tables

**Table 1:** Primer set for the detection of species of begomovirus and the identification of biotypes of *B. tabaci*.

Primers	Sequence (5′-3′)	Target	DNA Target (bp)	Target Region	References
***Begomovirus***					
PepYLCIV_4-F	ACCTTGGGGCTCAAGTCAAG	PepYLCIV	±997	DNA A Protein AC1	[20]
PepYLCIV_4-R	GGTCCCTATCTTTATAGTGGGCG				
TLCV-CPI	ATGGCGAAGCGACCAG	ToLCNDV	±771	DNA A Protein AV1	[21]
TLCV-CPT	TTAATTTGTGACCGAATCAT				
TYLCKaV-F	GTAACAGCCGAAGTGCACG	TYLCKaV	±1668	DNA B Protein BC1 and BV1	[22]
TYLCKaV-R	AATGGAGAGACACCAGTCTGCC				
**Biotypes of *B. tabaci***					
BaAF	GTGAAATCACTGTCCTCAGTTAGGT	biotype A	±812	DNA	[23]
BaAR	AAAGCCATAGACAAAGAAGTAGACG				
BaBF	CCACTATAATTATTGCTGTTCCCACA	biotype B	±661	Mitochondrial DNA mtCO1	
L2-N-3014R	TCCAATGCACTAATCTGCCATATTA				
BaANF	GGTTATTGCTGTTCCAACTGGG	biotype AN	±665	Mitochondrial DNA mtCO1	
L2-N-3014R	TCCAATGCACTAATCTGCCATATTA				
BaQF	GAAGCAACGCACTACTTACAA	biotype Q	±892, 700,400	DNA	
BaQR	TTCTCGGCGTTTTTACCAA				
BaNaF	GGCTTTGGTTTACTGGATTCTTTT	biotype Nauru	±578	Mitochondrial DNA mtCO1	
L2-N-3014R	TCCAATGCACTAATCTGCCATATTA				
BaSF	CTCGCAACATTGGGTGGTATAAAGT	biotype S	±613	Mitochondrial DNA mtCO1	
L2-N-3014R	TCCAATGCACTAATCTGCCATATTA				

**Table 2:** Observed symptoms, disease incidence, and disease severity of begomovirus infection on pepper in Java, Indonesia.

Location (District)	Altitude (m asl)	Observed Symptoms ^1^	Disease Incidence (%) *	Disease Severity (%) *
**Lowlands**				
Cirebon (West Java)	24	y, ym, lf, cp	86.00	29.50
Pangandaran 1 (West Java)	254	y, ym, gm, lf, cp, vb, st	93.00	57.50
Pangandaran 2 (West Java)	122	y, ym, gm, lf, cp, sl	93.00	45.75
Bantul (Yogyakarta)	9	y, ym, gm, lf, cp, vb	98.00	72.75
Kulon Progo (Yogyakarta)	11	y, ym, gm, lf, cp, vb, st	82.00	35.25
Sragen (Central Java)	163	y, ym, gm, lf, cp	97.00	60.00
Banyuwangi 1 (East Java)	141	y, ym, gm, lf, cp	90.00	44.75
Banyuwangi 2 (East Java)	108	y, ym, gm, lf, cp	98.00	63.75
Kediri (East Java)	176	y, ym, gm, lf, cp, vb, vc, st	100.00	81.25
**Mean**			**93.00 ^a^**	**54.50 ^a^**
**Highlands**				
Sukabumi (West Java)	872	y, ym, lf, cp	90.00	46.25
Bandung Barat (West Java)	1200	y, ym, gm, lf, cp	94.00	43.25
Garut (West Java)	1418	y, ym, gm, lf, cp	85.00	36.25
Banjarnegara (Central Java)	1167	y, ym, lf, cp, st	95.00	44.75
Wonosobo (Central Java)	1326	y, lf	69.00	22.50
Karanganyar (Central Java)	848	y, ym, lf, cp	88.00	31.75
Malang (East Java)	1185	y, ym, gm, lf, cp	94.00	49.50
Batu (East Java)	881	y, ym, gm, lf, cp	92.00	34.75
Magetan (East Java)	1024	y, ym, gm, lf, cp	92.00	34.00
**Mean**			**88.78 ^a^**	**38.11 ^b^**

^1^ y, yellowing; ym, yellow mosaic; gm, green mosaic; lf, leaf curl; cp, cupping; vb, vein banding; vc, vein clearing; st, stunting; sl, smaller leaf. * The means with different letters in the same column indicate a significant difference at a 5% level on *t*-test analysis.

**Table 3:** Biotypes of *B. tabaci* in pepper in lowlands and highlands in Java, Indonesia.

Location (District)	Biotype of *B. tabaci*					
	A	B	AN	Q	Nauru	S
**Lowlands**						
Cirebon (West Java)	+	-	-	+	-	-
Pangandaran 1 (West Java)	-	-	-	+	-	-
Pangandaran 2 (West Java)	-	+	-	-	-	-
Bantul (Yogyakarta)	-	+	-	-	-	-
Kulon Progo (Yogyakarta)	-	+	-	-	-	-
Sragen (Central Java)	-	+	-	-	-	-
Banyuwangi 1 (East Java)	-	+	-	-	-	-
Banyuwangi 2 (East Java)	+	-	-	-	-	-
Kediri (East Java)	+	-	+	-	-	-
**Highlands**						
Sukabumi (West Java)	-	-	-	+	-	-
Bandung Barat (West Java)	-	+	-	-	-	-
Garut (West Java)	-	-	-	-	-	-
Banjarnegara (Central Java)	-	+	-	-	-	-
Wonosobo (Central Java)	-	-	-	-	-	-
Karanganyar (Central Java)	-	-	-	-	-	-
Malang (East Java)	+	-	-	-	-	-
Batu (East Java)	-	+	-	-	-	-
Magetan (East Java)	-	-	-	+	-	-

+ and -, detected or not for those biotypes, respectively.

**Table 4:** Number and species of begomovirus infecting pepper among 20 diseased plants in lowlands and highlands in Java, Indonesia.

Location (District)	Results of PCR Detection								Percentage of Virus-Infected Plants (%) *
	No Detectable Product	Single Infection			Mixed Infections				
		PepYLCIV	ToLCNDV	TYLCKaV	PepYLCIV–ToLCNDV	PepYLCIV–TYLCKaV	ToLCNDV–TYLCKaV	PepYLCIV–ToLCNDV–TYLCKaV	
**Lowlands**									
Cirebon (West Java)		20							100.00
Pangandaran 1 (West Java)		18			2				100.00
Pangandaran 2 (West Java)		18				2			100.00
Bantul (Yogyakarta)		20							100.00
Kulon Progo (Yogyakarta)				11		6	1	2	100.00
Sragen (Central Java)	1	19							95.00
Banyuwangi 1 (East Java)	4	16							80.00
Banyuwangi 2 (East Java)	8	12							60.00
Kediri (East Java)	5	3			1	8		3	75.00
**Mean**									**90.00 ^a^**
**Highlands**									
Sukabumi (West Java)		15				5			100.00
Bandung Barat (West Java)		20							100.00
Garut (West Java)		20							100.00
Banjarnegara (Central Java)		19				1			100.00
Wonosobo (Central Java)	1	19							95.00
Karanganyar (Central Java)	1	19							95.00
Malang (East Java)	1	19							95.00
Batu (East Java)	1	19							95.00
Magetan (East Java)	9	10		1					55.00
**Mean**									**92.78 ^a^**

* The means with different letters in the same column indicate a significant difference at a 5% level on a *t*-test analysis.

## Data Availability

All data supporting this study are included as supplementary information.

## Supplementary Materials

The following supporting information can be downloaded at: https://www.mdpi.com/article/10.3390/v15061278/s1, Figure S1: Results of the amplification of the *B. tabaci* biotypes using 4 primers; Figure S2: Results of the amplification of the *begomovirus* species in Kediri-East Java using 3 primers.
