# Supplementary material for: Current Status and Complexity of Three Begomovirus Species in Pepper Plants in Lowlands and Highlands in Java Island, Indonesia
Source: Viruses. 2023 May 30;15(6):1278. doi: 10.3390/v15061278 (PMC10301231; doi:10.3390/v15061278)
Supplement: Supplementary file 1 [file viruses-15-01278-s001.zip › viruses-2405330-supplementary.pdf]

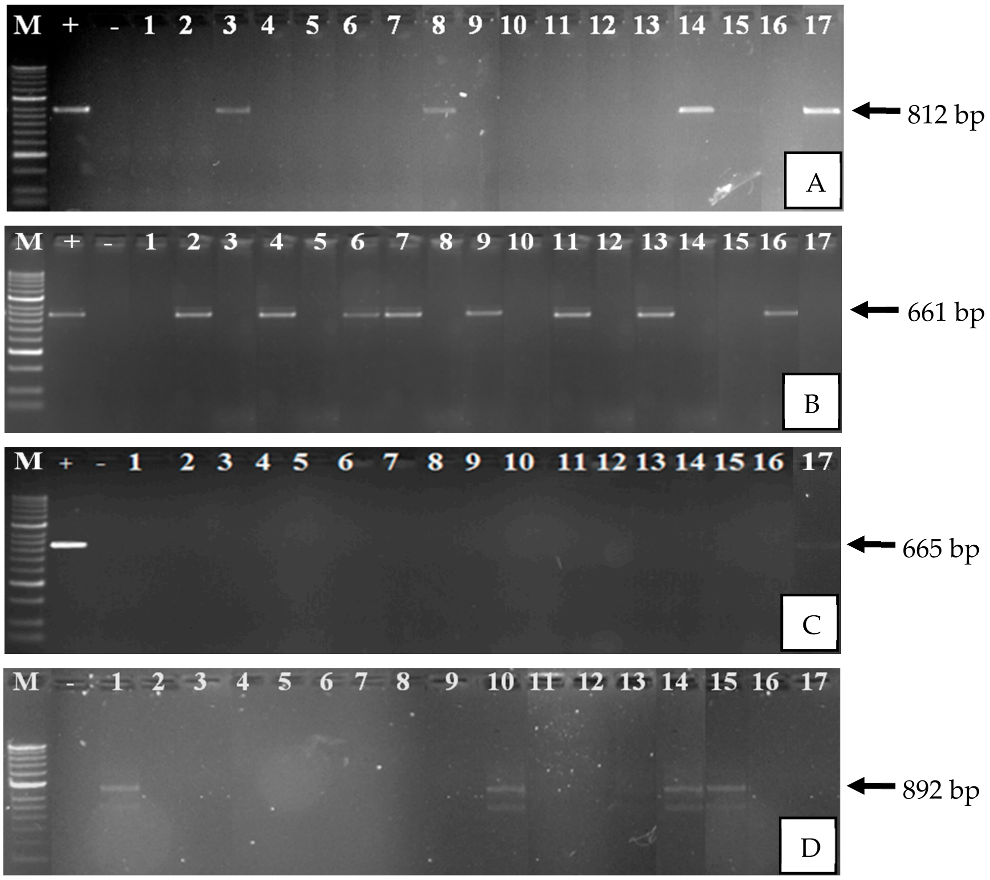

Figure S1. The results of amplification of the *B. tabaci* biotypes using 4 primers. (A) BaAF/BaAR (A biotype); (B) BaBF/ L2-N-3014R (B biotype); (C) BaANF/L2-N-3014R (AN biotype); (D) BaQF/BaQR (Q biotype). (M) 50bp hyper ladder Bioline; (+) positive control; (-) negative control/NTC; (1-17) samples of *B. tabaci*: (1) Sukabumi-West Java; (2) Banyuwangi 1-East Java; (3) Banyuwangi 2-East Java; (4) Banjarnegara-Central Java; (5) Wonosobo-Central Java; (6) Bantul-Yogyakarta; (7) Kulonprogo-Yogyakarta; (8) Malang-East Java; (9) Batu-East Java; (10) Magetan-East Java; (11) Sragen-Central Java; (12) Garut-West Java; (13) Bandung Barat-West Java; (14) Cirebon-West Java; (15) Pangandaran 1-West Java; (16) Pangandaran 2-West Java; (17) Kediri-East Java.

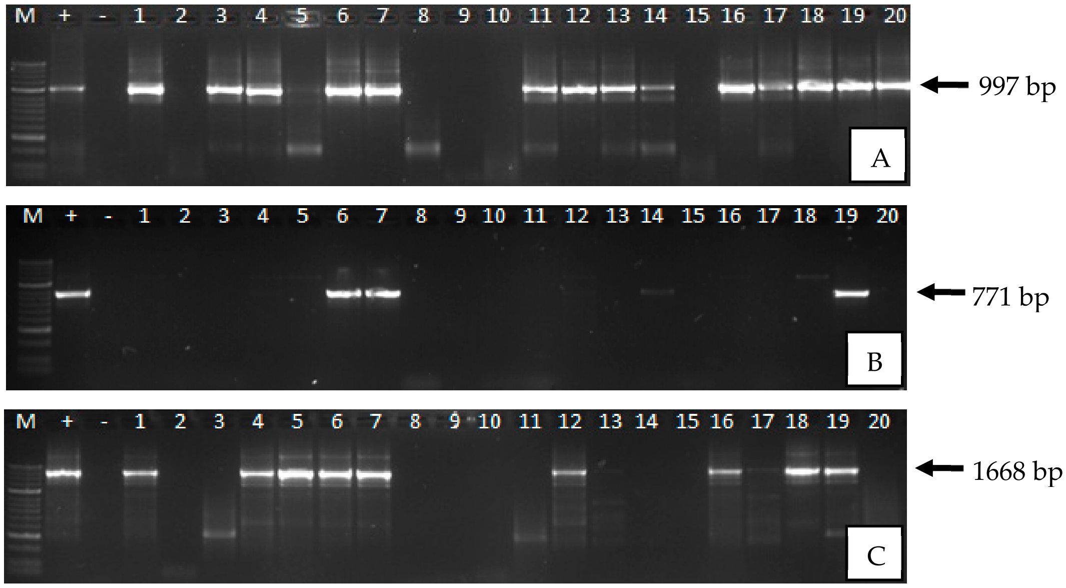

Figure S2. The results of amplification of the *begomovirus* species in Kediri-East Java using 3 primers. (A) PepYLCIV-F/R (PepYLCIV); (B) ToLCNDV-F/R(ToLCNDV); (C) TYLCKaV-F/R (TYLCKaV). (M) 50bp hyper ladder Bioline; (+) positive control; (-) negative control/NTC; (1-20) leaf samples.
